# Strategies for population-level identification of post-acute sequelae of COVID-19 through health administrative data

**DOI:** 10.3389/fpubh.2025.1637112

**Published:** 2025-08-20

**Authors:** Cristina Mazzali, Pietro Magnoni, Alberto Zucchi, Giovanni Maifredi, Luca Cavalieri d’Oro, Maria Letizia Gambino, Anna Clara Fanetti, Pietro Giovanni Perotti, Marco Villa, Maria Grazia Valsecchi, Daria Vigani, Claudio Lucifora, Antonio Giampiero Russo

**Affiliations:** ^1^Epidemiology Unit, Agency for Health Protection Milan, Milan, Italy; ^2^Epidemiology Unit, Agency for Health Protection Bergamo, Bergamo, Italy; ^3^Epidemiology Unit, Agency for Health Protection Brescia, Brescia, Italy; ^4^Epidemiology Unit, Agency for Health Protection Brianza, Monza, Italy; ^5^Epidemiology Unit, Agency for Health Protection Insubria, Varese, Italy; ^6^Epidemiology Unit, Agency for Health Protection Montagna, Sondrio, Italy; ^7^Epidemiology Unit, Agency for Health Protection Pavia, Pavia, Italy; ^8^Epidemiology Unit, Agency for Health Protection Val Padana, Cremona, Italy; ^9^School of Medicine and Surgery and Bicocca Bioinformatics Biostatistics and Bioimaging Centre (B4), University of Milano-Bicocca, Milan, Italy; ^10^Department of Economics and Finance, Catholic University of the Sacred Heart, Milan, Italy

**Keywords:** COVID-19, PASC, long COVID, health administrative data, routinely collected data, case-detection algorithm

## Abstract

**Introduction:**

Post-acute sequelae of COVID-19 (PASC) encompass several clinical outcomes, from new-onset symptoms to both acute and chronic diagnoses, including pulmonary and extrapulmonary manifestations. Health administrative data (HAD) from health information systems allow population-level analyses of such outcomes. Our primary aim was to identify clinical conditions potentially attributable to SARS-CoV-2 infection, and the types of HAD and “diagnostic criteria” used for their detection.

**Methods:**

We performed a literature review to identify HAD-based cohort studies assessing the association between SARS-CoV-2 infection and medium−/long-term outcomes in the general population. From each included study, we extracted data on design, algorithms used for outcome identification (sources, coding systems, codes, time criteria/thresholds), and whether significant associations with SARS-CoV-2 infection were reported.

**Results:**

We identified six studies investigating acute and chronic conditions grouped by clinical domain (cardiovascular, respiratory, neurologic, mental health, endocrine/metabolic, pediatric, miscellaneous). Two studies also addressed the onset of specific symptoms. Cardio/cerebrovascular conditions were most studied, with significant associations reported for deep vein thrombosis, heart failure, atrial fibrillation, and coronary artery disease. Conditions in other domains were less investigated, with inconsistent findings. Only three studies were designed as test-positive vs. test-negative comparisons.

**Discussion:**

Heterogeneity in data sources, study design, and outcome definitions hinder the comparability of studies and explain the inconsistencies in findings about associations with SARS-CoV-2 infection. Rigorously designed studies on large populations with wide availability of data from health information systems are needed for population-level analyses on PASC, and especially on its impact on chronic diseases and their future burden on healthcare systems.

## Introduction

A growing body of evidence suggests that SARS-CoV-2 infection and its resulting disease may lead to sequelae persisting beyond the typical post-viral recovery period (1, 2). This phenomenon is referred to by a variety of terms, such as chronic COVID-19 syndrome, late sequelae of COVID-19, long COVID, long-haul COVID, long-term COVID-19, post-COVID syndrome, post-acute COVID-19, and post-acute sequelae of SARS-CoV-2 infection (PASC). To harmonize the discrepancies in nomenclature, the World Health Organization (WHO) proposed the term Post-COVID-19 Condition (PCC). PCC is defined as the continuation or the development of new symptoms 3 months after a probable or confirmed SARS-CoV-2 infection, where these symptoms last for at least 2 months and cannot be explained by an alternative diagnosis (3).

Post-COVID-19 conditions encompass a wide range of clinical outcomes, spanning from the emergence of new symptoms to both acute and chronic clinical diagnoses. In a 2021 study, Al-Aly et al. (1) identified an extensive array of sequelae within 6 months among individuals surviving at least 30 days from symptom onset. These included both pulmonary and extrapulmonary manifestations, such as neurological and neurocognitive disorders, mental health conditions, metabolic, cardiovascular, and gastrointestinal disorders, as well as general malaise, fatigue, musculoskeletal pain, and anemia. Estiri et al. (2) extended the period of observation, investigating symptoms and conditions up to 9 months following infection. Many studies have focused on hospitalized COVID-19 patients, limiting the generalizability of findings to broader populations. Several authors have argued about the need to investigate clinical sequelae in low-risk adult populations, or in individuals who experienced mild or asymptomatic infections (4). Currently, an increasing number of studies assesses these conditions at the population level, or at least across large regional areas or specific population subgroups (5–10).

Identifying health outcomes on a population level in a timely, systematic, and cost-efficient manner is crucial for implementing effective public health strategies. Health administrative data (HAD), routinely generated through the provision of health services, provide a valuable resource for this purpose. Although labeled “administrative,” these data are primarily produced within national, regional or local health information systems, and reflect both clinical and service use information. HAD-based detection algorithms to be applied to the general population can be developed by linking multiple data sources, such as billing claims, hospital discharge records, outpatient specialist services, pharmaceutical prescriptions, emergency department visits, general practitioner records and co-payments exemption data, at the individual level. The specific context of application, the quality and availability of administrative data and the extent to which different datasets can be linked strongly affect the possibility of examining isolated symptoms, acute conditions, or chronic diseases.

Recent critiques of the existing literature have highlighted methodological limitations, especially the lack of standardized study designs and the use of limited comparative methods (5). Many studies, for example, do not include SARS-CoV-2-negative individuals as a control group, limiting the ability to disentangle the effects of infection from the effect of other specific disease progressions or conditions (10). Further research is needed to investigate the potential protective role of vaccination against long-term consequences of SARS-CoV-2 infection (5).

The primary objective of the present study is to identify, via a literature review, clinical conditions potentially attributable to SARS-CoV-2 infection, and the types of health administrative data and “diagnostic criteria” used for population-level investigations of these outcomes. We also aim to analyze the study designs employed for examining medium- and long-term sequelae, comparing infected and non-infected individuals.

## Methods

**Figure 1:**
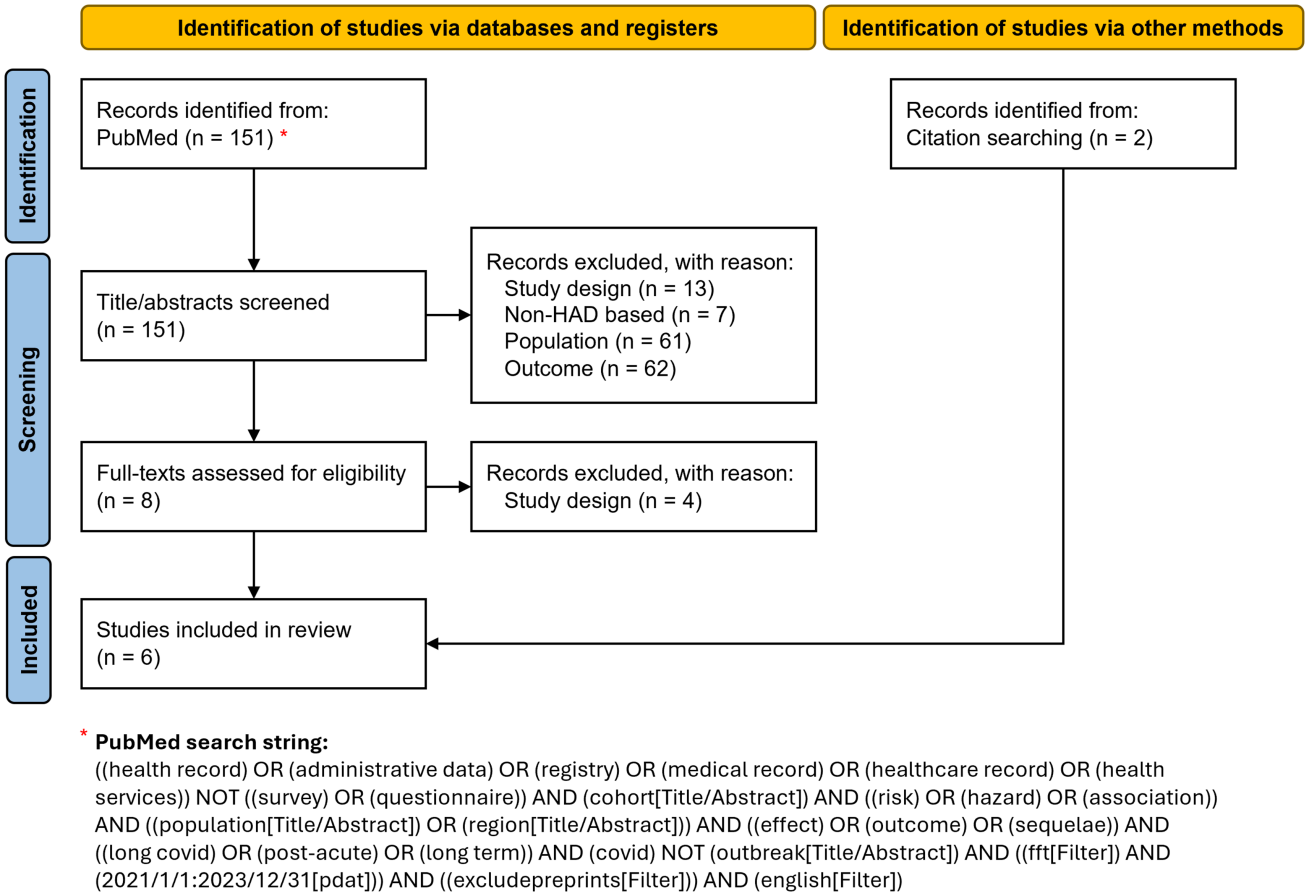
Study selection flowchart illustrating the identification, screening, eligibility assessment, and inclusion of studies in the review. Adapted from Page et al. (11).

A narrative literature review was conducted to identify comparative cohort studies based on HAD that examine medium- and long-term outcomes of SARS-CoV-2 infection in the general population, to assess the effects of infection. A structured PubMed search, updated in March 2025, was conducted using a Boolean combination of terms related to health administrative data, cohort study design, population-level analyses, and PCC-related outcomes. Filters were applied to restrict results to English-language studies published between 2021 and 2023. The full search string is detailed in Figure 1 and in Supplementary Table S1. Additional relevant studies were identified through reference screening of included articles.

We focused on primary, comparative, non-descriptive cohort studies based on administrative data for the investigation of specific medium- or long-term clinical outcomes. Therefore, we applied the following exclusion criteria: patient-centered studies based on surveys or laboratory data; case–control studies; studies that developed prognostic models; studies focusing solely on healthcare resource utilization as outcomes; studies examining the impact of COVID-19 on healthcare service delivery; studies performed on selected population subgroups (e.g., veterans) instead of the general population; studies evaluating the effect of specific risk factors, therapeutic interventions and/or vaccination.

From each selected study, the research protocol and methods used to identify potential outcomes through HAD were retrieved. Algorithms used for outcome identification may or may not involve linkages across different data sources. We then extracted information about the health information system source(s), coding system(s), specific diagnostic or medication code(s), and time criteria/thresholds used to identify outcomes.

## Results

A total of 151 articles were initially identified via PubMed. Of these, four (5–8) met our inclusion criteria. Two more studies (9, 10) were identified through reference screening and included in our review (Figure 1; Table 1). The diagnosis and/or medication codes used for the identification of each outcome by the six studies are listed in Supplementary Tables S2–S6.

**Table 1:** Study characteristics of the six literature results included in the review.

Authors	Publication year	Country	Population size	Data source(s)
Mizrahi et al. (5)	2023	Israel	1,913,234	EHR from Maccabi Healthcare Services
Lund et al. (6)	2021	Denmark	526,406	Danish national health registries, Danish COVID-19 cohort
Lam et al. (7)	2023	Hong Kong; UK	7,700,806 (HK); 502,616 (UK)	EMRs from Hong Kong Hospital Authority (HKHA); United Kingdom BioBank, Primary care (GP) records from the Phoenix Partnership (TPP) and Egton Medical Information Systems (EMIS) Health GP system of England; hospital inpatient data from National Health Service (NHS) Digital; national death registry; diagnostic COVID-19 test results from Public Health England (PHE), Public Health Scotland (PHS) and Secure Anonymized Information Linkage (SAIL)
Wan et al. (8)	2023	UK	502,476	United Kingdom BioBank, Primary care (GP) records from the Phoenix Partnership (TPP) and Egton Medical Information Systems (EMIS) Health GP system of England; hospital inpatient data from National Health Service (NHS) Digital; national death registry; diagnostic COVID-19 test results from Public Health England (PHE), Public Health Scotland (PHS) and Secure Anonymized Information Linkage (SAIL)
Naveed et al. (9)	2023	Canada	629,935	British Columbia COVID-19 Cohort integrating COVID-19 data sets (including testing, case, hospitalization, and vaccination data) with registry and administrative data
Horberg et al. (10)	2022	USA	31,390	EMRs from Kaiser Permanente Mid-Atlantic States databases

### Details of individual studies

Mizrahi et al. (5) grouped several potential short- and long-term effects of COVID-19 into four categories: symptoms, new diagnoses of chronic diseases, new acute complications, and new infectious diseases. Outcomes were further classified as either recurrent or first-time events (Supplementary Table S2). The study analyzed electronic health records (EHRs) from the Maccabi Healthcare Services database, the second-largest health fund in Israel. All individuals with a COVID-19 test between March 1, 2020, and October 1, 2021, were included. Patients who were hospitalized within 30 days of infection were excluded in order to focus on mild cases. Available data included diagnoses, chronic diseases, billing codes, dispensed medications, and laboratory data. Outcomes were identified using ICD-10 coded diagnoses recorded in EHRs. For pulmonary outcomes, severity was assessed through prescribed medications for obstructive airway diseases (ATC code R03).

Lund et al. (6) considered these outcomes: delayed acute complications, onset of chronic diseases, persistent symptoms, initiation of prescriptions potentially associated with delayed complications. Their study also evaluated overall healthcare utilization, i.e., visits to general practitioners, outpatient services, emergency department visits, and hospitalizations (Supplementary Table S3). The cohort included the Danish population from February 27 to May 31, 2020. New prescriptions were identified using the Danish National Prescription Registry. Diagnoses related to delayed complications, new chronic conditions, or persistent symptoms were obtained from inpatient and outpatient data in the Danish National Patient Registry (ICD-10). For acute kidney disease, laboratory creatinine values were used.

The multi-database study by Lam et al. (7) used inpatient data from the Hong Kong Hospital Authority (HKHA) and inpatient plus outpatient data from the UK Biobank (UKB). Patients were enrolled between April 1, 2020 (HKHA) or March 16, 2020 (UKB), and May 31, 2021. Outcomes were measured as incidence rates for: myocardial infarction, heart failure, stroke, atrial fibrillation, coronary artery disease, deep vein thrombosis, interstitial lung disease, acute respiratory distress syndrome, chronic pulmonary disease, seizure, Bell’s palsy, encephalitis and encephalopathy, anxiety, post-traumatic stress disorder, psychotic disorder, liver injury, pancreatitis, acute kidney injury, end-stage renal disease. UKB used ICD10 coding, whereas ICD-9-CM codes were used for outcome identification from hospitalization data of HKHA (Supplementary Table S4). Additional outcomes included: major cardiovascular diseases (composite outcome of stroke, heart failure, and coronary heart disease); cardiovascular mortality; all-cause mortality.

In Wan et al. (8), the cohort of subjects with an infection (March 16—November 30, 2020) was compared with two control cohorts: a contemporary uninfected group (March 16, 2020—August 31, 2021) and a historical cohort (March 16—November 30, 2018). The study focused on: major cardiovascular diseases (composite of heart failure, stroke, coronary heart disease); stroke; transient ischemic attack (TIA); atrial fibrillation; atrial flutter; pericarditis; myocarditis; coronary heart disease; acute coronary syndrome; myocardial infarction; ischemic cardiomyopathy; stable angina; unstable angina; heart failure; non-ischemic cardiomyopathy; cardiac arrest; cardiogenic shock; deep vein thrombosis; superficial vein thrombosis; cardiovascular mortality; all-cause mortality. Outcome identification relied on inpatient hospital data and general practitioner records via ICD-10 codes (Supplementary Table S5).

The study by Naveed et al. (9) focused on the association between COVID-19 infection and the onset of diabetes. The study included all individuals tested for COVID-19 in British Columbia between January 1, 2020, and December 31, 2021. Diabetes was identified using an algorithm applied to medical visit records, hospitalizations, chronic disease registries, and prescriptions of diabetes-specific medications. A subject was classified as diabetic if any of the following criteria were met: two medical visits with ICD-9-CM code 250.xx within 1 year (Medical Service Plan); hospital admission with a diabetes-related code (ICD-9-CM 250.xx or ICD-10-CA E10*–E14*); prescription of at least two oral hypoglycemic agents or insulin within 1 year.

Finally, Horberg et al. (10) adopted a different approach. They used data from the Kaiser Permanente Mid-Atlantic States (KPMAS) program, which includes information on primary and specialist care, outpatient services, and hospitalizations. The study included all KPMAS patients tested for COVID-19 between January 1, 2020, and December 31, 2021. COVID-19–positive patients’ diagnoses in the post-infection period were extracted and grouped using the Clinical Classification Software (CCS) developed within the HCUP project. The “Category” aggregation level was used to maintain sufficient specificity for identifying distinct conditions. Some modifications were made manually following consultation with infectious disease experts. To determine which CCS conditions could indicate potential PASC, the proportion of patients with a specific CCS condition was calculated over the total number of patients with any CCS diagnosis within a given timeframe. Three timeframes were defined based on the test date (T0): diagnosis within the 4 years preceding T0 (*pre-existing condition*); diagnosis occurring within 30 days from T0 and persisting through 120 days (*acute and persistent condition*); diagnosis occurring between 30 and 120 days from T0 (*subsequent condition*). An aggregate percentage across all time frames was compared against a 0.04% empirical threshold. Remaining diagnoses were reviewed by clinicians to assess the biological plausibility of their association with PASC. Conditions identified as potential PASC are listed in Supplementary Table S6.

Supplementary Table S7 presents a comparison of ICD-9 and ICD-10 diagnosis codes used to identify symptoms and conditions in the first five studies reviewed (5–9). All studies used ICD-10, with two also incorporating ICD-9 coding.

### Synthesis

Based on the studies analyzed, a preliminary distinction can be made between algorithms used to identify acute or chronic conditions and those used to identify isolated symptoms. The acute and chronic conditions identified, grouped by clinical domain, fall into the following categories: cardiovascular, respiratory, neurologic, mental health, endocrine/metabolic, pediatric, miscellaneous. Symptoms were specifically investigated in two studies: more comprehensively in Mizrahi et al. (5) and in a more limited way in Lund et al. (6).

Almost all studies used multiple data sources to identify health conditions. Hospitalization data were used in all studies and served as the sole data source in one case (7). Other sources included specialist medical visits, general practitioner databases, prescription records, emergency department visits, and disease registries. In one study (5), comprehensive electronic health records (EHRs) were used.

**Figure 2:**
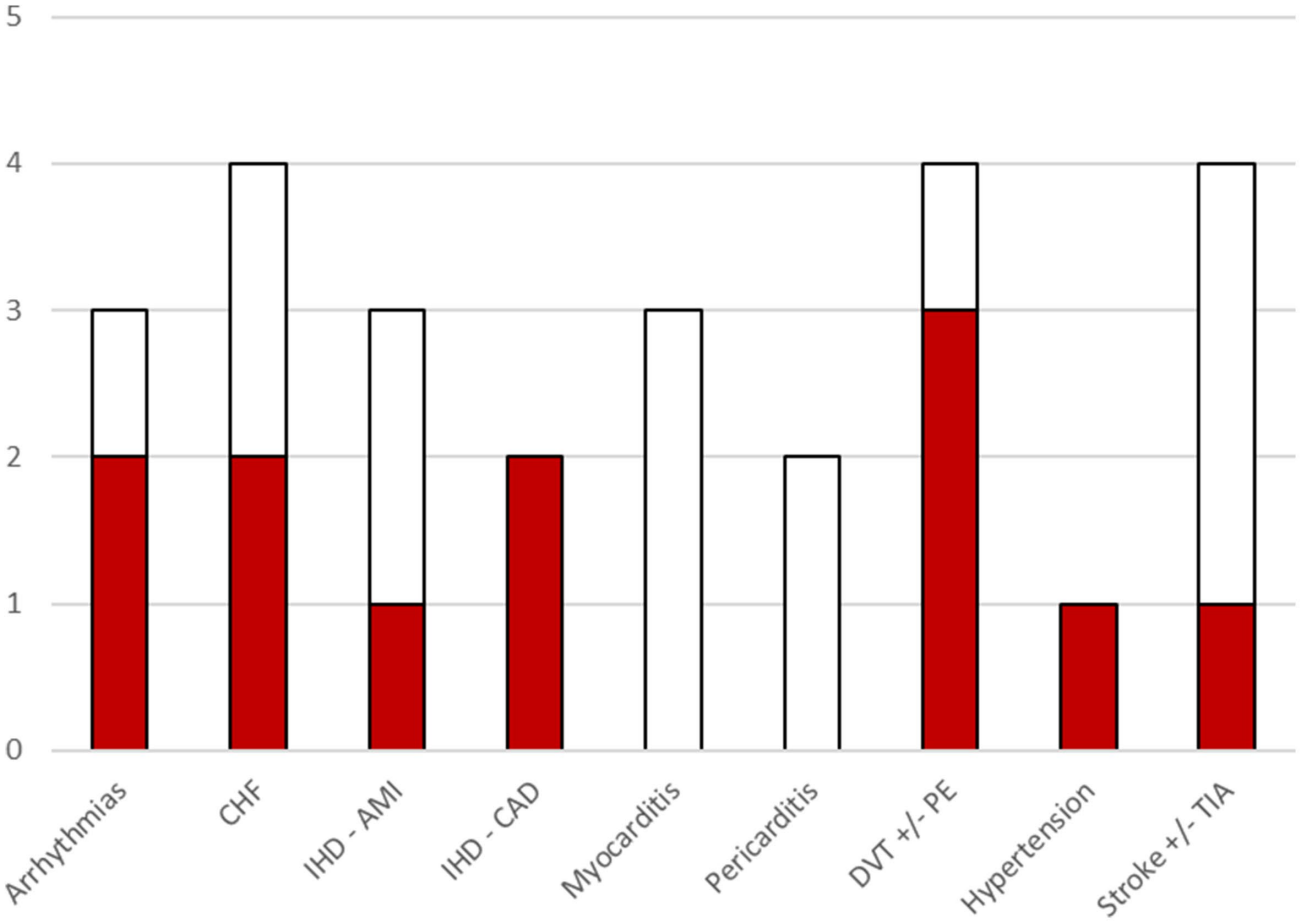
Bar chart illustrating, for each cardio/cerebrovascular outcome, the number of included studies that investigated that outcome. The colored portion of each bar indicates the number of included studies where a significant association of the outcome with COVID-19 was found. Within the category arrhythmias, associations were found for atrial fibrillation. CHF, Congestive Heart failure; IHD, Ischemic heart disease; AMI, acute myocardial infarction; CAD, coronary artery disease; DVT, Deep vein thrombosis; PE, pulmonary embolism; TIA, transient ischemic attack.

Cardiovascular and cerebrovascular conditions were the most frequently studied, although their definitions were not consistent across studies. The conditions most often associated with SARS-CoV-2 infection were deep vein thrombosis, congestive heart failure, atrial fibrillation, and coronary artery disease (Figure 2). Regarding respiratory conditions, acute respiratory distress syndrome (ARDS) was examined in two studies, with one reporting a significant association with infection. Interstitial lung diseases (particularly pulmonary fibrosis) were analyzed in three studies; one of these, which used broader condition definitions, found a significant association. Chronic pulmonary diseases were also studied at various levels of aggregation and were found to be significantly associated with infection. Neurological conditions were identified using various groupings of diagnostic codes. Encephalitis was investigated in three studies, with none reporting significant associations. Epilepsy was studied in two studies, with one finding a significant association with infection. Among mental health conditions, anxiety, depression, psychosis, and broader psychiatric disorders were examined. Anxiety was studied in three papers and found to be significantly associated with SARS-CoV-2 infection in two. Psychiatric disorders were aggregated differently across studies but showed significant differences between infected and non-infected individuals in two cases. Diabetes mellitus was analyzed in three studies, sometimes without differentiating between type 1 and type 2. A significant association with SARS-CoV-2 infection was reported in Naveed et al. (9), where diabetes was specifically investigated. Two studies focused on pediatric conditions, particularly Kawasaki disease and pediatric multisystem inflammatory syndrome, but no significant associations were found.

All reviewed studies were, by design, comparative cohort studies aimed at examining the association between SARS-CoV-2 infection (or COVID-19 specifically) and potential sequelae in a population using routinely collected data. However, not all studies included a comparison between test-positive and test-negative (for SARS-CoV-2 infection) subjects as part of their study design (7, 8). In contrast, Horberg et al. (10) employed a fundamentally different approach: rather than investigating predefined conditions, the study broadly assessed health conditions among both COVID-positive and COVID-negative individuals to identify those potentially associated with infection. Details of methodologies of the remaining three studies (5, 6, 9) are summarized in Table 2.

**Table 2:** Comparison of methods for test-positive vs. negative analyses.

Item	Mizrahi et al. (5)	Lund et al. (6)	Naveed et al. (9)
Cohort: positive cases	Subjects with positive PCR test result	Subjects with positive PCR test result not hospitalized within 2 weeks from test date	Subjects with positive PCR test result
Cohort: controls	Subjects with negative PCR test result	1st group: subjects with negative PCR test result not hospitalized; 2nd group: subjects with positive test hospitalized on test day or within 2 weeks, discharged alive and not readmitted within 2 weeks	Subjects with negative PCR test result
Enrollment period	Tests performed between 03/01/2020 and 10/01/2021	Tests performed between 02/27/2020 and 05/31/2020	Tests performed between 01/01/2020 and 12/31/2021
Exclusion criteria	Subjects followed by the health organization for less than 1 year	Subjects residing less than 1 year in Denmark, inconclusive tests, deceased within 2 weeks after test execution. Excluded from second control group: subjects discharged after 05/31/2020	Subjects < 18 years old; history of diabetes or new diagnosis of diabetes within 30 days of a positive test; deceased within 30 days of the positive test; long-term care residents
Index event and matching	First positive test for exposed subjects. A negative test not preceded by a positive test for controls. If during follow-up a non-exposed (negative) subject becomes infected, the pair is censored. The negative subject re-enters the study as exposed (positive) and is matched to a new non-exposed (negative) subject. Exact 1:1 matching by birth year, sex, test month, and COVID-19 immunization status at test date	Matching 1:10 with the first control group, random without replacement, exact for birth year, sex, and test week.	First positive test for exposed subjects. A negative test not preceded by a positive test for controls. A single test was randomly selected for non-exposed subjects who had more than one negative test. Matching 1:4 on sex (exact), age (± 3 years), test date (± 7 days)
Outcome	Incidence of: symptoms, new diagnoses of chronic diseases, new acute complications, and new infectious diseases	Delayed acute complications, chronic diseases, persistent symptoms, and medication prescriptions	Incident diabetes identified more than 30 days after the index date
Comparisons	Compared outcomes between unvaccinated positive and unvaccinated negative subjects. Additionally, compared outcomes between unvaccinated positive and vaccinated positive subjects.	In the main analysis, compared positive and negative test subjects.	In the main analysis, compared positive and negative test subjects.
Follow-up period	From second to 12 months after the test (index date)	From 2 to 6 months after test execution	From 30 days to study closure date (01/31/2022)
Censoring	Follow-up ends at the earliest of: study period end, death, withdrawal from the health program, second infection of the exposed subject (new positive test > 90 days after the first), or infection of the non-exposed subject.	Follow-up ends at the earliest of: study period end and death.	Follow-up ends at the earliest of: study period end and death.
Statistical analysis and association measures	Weighted Cox model via inverse propensity score. Association assessed through hazard ratio in the 30–180 days and 180–360 days periods. Risk differences assessed via weighted Kaplan–Meier in the 30–180 days, 30–360 days, and 180–360 days periods.	Risk ratios calculated for different observation periods: pre-test (6 months to 2 weeks before the SARS-CoV-2 test) and follow-up (2 weeks to 6 months after the test) using a Poisson model.	Cumulative incidence curves were generated using Kaplan–Meier method. Diabetes risk in both groups was compared using Cox model. The population fraction attributable to COVID-19 adjusted for confounders was assessed using Cox model.
Adjustment	Propensity score calculated via logistic regression considering: pre-existing specific chronic conditions, alcohol consumption, smoking habits, ethnicity or social group, socioeconomic status, influenza vaccination history from the past 3 years.	Confounding controlled using weighted estimates via propensity score, with negative subjects weighted by propensity odds and negative subjects with weight 1. Pre-specified confounders used for score calculation.	Chronic conditions considered include: acute myocardial infarction, asthma, chronic kidney disease, chronic liver disease, COPD, depression, and hypertension. Additionally, glucocorticoid use, alcohol abuse, and injection drug use were considered. Also considered vaccination status and socioeconomic status.

## Discussion

The studies reviewed provide multiple lines of evidence supporting the presence of medium- and long-term conditions potentially associated with SARS-CoV-2 infection or COVID-19. The conditions investigated in the literature cover a wide spectrum, ranging from symptoms to acute and chronic diseases, and extend beyond the respiratory system to various other organs and systems.

Conducting research at the population level requires the use of routinely collected data and the capacity to link different data sources in order to reconstruct individual subjects’ clinical histories. The data sources used are diverse and include general practitioner or specialist visits, outpatient diagnoses, hospital admissions, pharmaceutical prescriptions, and, to a lesser extent, laboratory data. The availability, completeness and quality of data directly influence the scope and extent of studies leveraging them. In the absence of coded diagnoses from general practitioner or specialist visits, it is virtually impossible to investigate non-specific symptoms such as fatigue or cough. Similarly, acute conditions are more likely to be detected through significant and specific healthcare encounters, such as hospitalizations. For less severe conditions, detection may be less sensitive. Chronic conditions, on the other hand, are more likely to be captured even with less detailed data, particularly when multiple sources can be integrated.

Several studies define the exposure window for participant inclusion based on the period during which different viral variants were dominant. Some studies do not address the influence of variant dominance in comparing test-positive and test-negative individuals, while others account for it in the analyses. Few studies limit the enrolment period to phases with minimal overlap between circulating variants, to reduce confounding.

The follow-up periods used to identify post-infection sequelae vary widely across studies. Typically, a lag of 3 weeks to 30 days post-infection is used to separate acute complications. Observation periods range from four to 12 months, although some studies extend beyond 1 year.

Possibly due to limited availability of tests during certain phases of the pandemic, many studies do not directly compare test-positive versus test-negative individuals. In some cases, the exposed group includes individuals with a positive test as well as those hospitalized for conditions consistent with COVID-19 without test confirmation. Conversely, the unexposed group is sometimes defined generically as individuals without a positive test result. These definitions may reduce the robustness of comparisons and limit the interpretation of associations.

The most frequently reported measures of association are hazard ratios and risk differences, typically estimated using Cox proportional hazards models or Kaplan–Meier methods, adjusted for confounders. Inverse probability weighting is often used to achieve covariate balance. Common confounders include comorbidities—often specific to the outcome under investigation—along with alcohol and tobacco use and sociodemographic characteristics.

## Conclusion

The evidence emerging from the studies analyzed confirms that SARS-CoV-2 infection can lead to medium- and long-term clinical consequences, ranging from non-specific symptoms to acute and chronic diseases. Nevertheless, our understanding of these sequelae remains limited and fragmented, primarily due to heterogeneity in data sources, coding practices, and methodological designs.

The integrated use of data from health information systems offers remarkable potential for investigating the long-term consequences of the infection, as it enables the analysis of large populations—including individuals with severe disease as well as those with mild or asymptomatic infections. However, the variability in data availability and quality—along with country-specific differences in coding systems, healthcare organization, and care pathways—poses challenges to harmonizing results. In particular, the lack of precise coding in primary care can impede the early and accurate identification of post-COVID manifestations such as fatigue or cough. Likewise, for rare or milder conditions, the detection rate may decrease if there is no systematic referral to specialist consultations or hospital care.

There is an evident need to include test-negative control groups or adopt appropriate comparison strategies in order to distinguish the effects attributable to infection from those related to the natural progression of other conditions. A further step forward would be to incorporate analyses that consider the influence of protective factors, such as vaccination, as well as the role of different viral variants.

Addressing these challenges requires large-scale, multicenter studies that employ rigorous methodologies, leveraging the potential of administrative data while standardizing coding tools and outcome definitions. An integrated approach—combining clinical data, disease registries, pharmaceutical prescriptions, exemptions, and electronic medical records—can provide a more complete picture of the post-infection trajectory, ultimately guiding public health policies and service planning toward the management and prevention of long-term complications.

In conclusion, although current findings support the existence of a broad range of post-COVID sequelae, further research with robust protocols and large cohorts is essential. Only through such efforts will it be possible to fully quantify the impact of so-called “long COVID” and provide clear guidance on prevention, early diagnosis, and long-term care, with particular emphasis on the emergence of new chronic diseases. Even after the pandemic emergency phase, these conditions will continue to exert a significant influence on healthcare systems worldwide.
