# Supplementary material for: Strategies for population-level identification of post-acute sequelae of COVID-19 through health administrative data
Source: Front Public Health. 2025 Aug 20;13:1637112. doi: 10.3389/fpubh.2025.1637112 (PMC12405178; doi:10.3389/fpubh.2025.1637112)
Supplement: Supplementary file 1 [file Data_Sheet_1.pdf]

Strategies for population-level identification of Post-Acute Sequelae of COVID-19 through health administrative data

Cristina Mazzali (1), Pietro Magnoni (1) *, Alberto Zucchi (2), Giovanni Maifredi (3), Luca Cavalieri d'Oro (4), Maria Letizia Gambino (5), Anna Clara Fanetti (6), Pietro Giovanni Perotti (7), Marco Villa (8), Maria Grazia Valsecchi (9), Daria Vigani (10), Claudio Lucifora (10), Antonio Giampiero Russo (1); *on behalf of the PASCNET study group*

Affiliations

1. Epidemiology Unit, Agency for Health Protection Milan, Milan, Italy
2. Epidemiology Unit, Agency for Health Protection Bergamo, Bergamo, Italy
3. Epidemiology Unit, Agency for Health Protection Brescia, Brescia, Italy
4. Epidemiology Unit, Agency for Health Protection Brianza, Monza, Italy
5. Epidemiology Unit, Agency for Health Protection Insubria, Varese, Italy
6. Epidemiology Unit, Agency for Health Protection Montagna, Sondrio, Italy
7. Epidemiology Unit, Agency for Health Protection Pavia, Pavia, Italy
8. Epidemiology Unit, Agency for Health Protection Val Padana, Cremona, Italy
9. University of Milano-Bicocca, School of Medicine and Surgery and Bicocca Bioinformatics Biostatistics and Bioimaging Centre (B4), Milan, Italy
10. Department of Economics and Finance, Catholic University of the Sacred Heart, Milan, Italy

Corresponding author

Pietro Magnoni. Epidemiology Unit, Agency for Health Protection (ATS) of Milan, Via Conca del Naviglio 45, 20123 Milan, Italy. E-mail: pmagnoni@ats-milano.it

Supplementary Material

Supplementary Table S1. Full PubMed search string constructed by combining sections pertaining to health administrative data, cohort study design, population-level analyses, and post-COVID condition (PCC) outcomes for this review.

**Health administrative data**	((health record) OR (administrative data) OR (registry) OR (medical record) OR (healthcare record) OR (health services)) NOT ((survey) OR (questionnaire))
	AND
**Cohort study design**	(cohort[Title/Abstract]) AND ((risk) OR (hazard) OR (association))
	AND
**Population-level analyses**	((population[Title/Abstract]) OR (region[Title/Abstract]))
	AND
**PCC-related outcomes**	((effect) OR (outcome) OR (sequelae)) AND ((long covid) OR (post-acute) OR (long term)) AND (covid)
	NOT (outbreak[Title/Abstract])
	AND
**Additional filters**	((fft[Filter]) AND (2021/1/1:2023/12/31[pdat])) AND ((excludepreprints[Filter])) AND (english[Filter])

Supplementary Table S2 (A-D). List of outcomes in the categories "Symptoms" (A), "Chronic conditions" (B), "Acute diagnoses" (C), "Infectious diseases" (D) investigated by Mizrahi et al. (5). Conditions that were not considered in comparative analyses between COVID-positive and COVID-negative subjects due to insufficient numbers of events are highlighted in gray.

**A. Symptoms**			
**Outcome**	**Subcategory**	**ICD-10**	**First occurrence**
Abdominal pain		R10.9	
Anosmia and Dysgeusia		R43.0, R43.8	
Arthralgia		M25.5	
Bloody stool		K92.1	
Chest pain		R07.9, R07.1, R07.8, R07.2	
Chills		R68.83	
Concentration and memory impairment	Memory difficulties	R41.83, R41.2, R68.89	
	ADHD	F90.0 (314.00, Y23257)	
	Cognitive deficit	R41.83, R41.2	
Cough		R05	
Convulsions		R56.9, R56.01, R56.00	1
Diarrhea		R19.7	
Dizziness		R42	
Dyspnea		R06	
Hair loss		L65.8, L65.0, L65.9	
Headache		G44, G43	
Hyperhidrosis		R61	
Insomnia		F51	
Loss of appetite		R63.4, R63.6	
Lymphadenopathy		R59.9	
Menstruation abnormalities		N91, N92	1
Muscle atrophy		M62.5	
Myalgia		M60.9	
Nausea or vomiting		R11.0, R11.2, R11.10	
Palpitations		R00.0, R00.2	
Paresthesia		R20	

A. Symptoms			
**Outcome**	**Subcategory**	**ICD-10**	**First occurrence**
Respiratory disorders		J98 (excluded J98.1, J98.5, J98.6, J98.8, J98.01)	1
Rhinorrhea		J34.8	
Skin rash		R21, R50.6, R53.9	
Sore throat		R07.0	
Tinnitus		H93	
Tremor		R25.9	
Visual disturbances		H53	
Voice disorder		R49.9	
Weakness		R53	
Weight loss		R63.4	

**B. Chronic conditions**			
**Outcome**	**Subcategory**	**ICD-10**	**First occurrence**
Anxiety		F41	1
Cardiac arrhythmias	Paroxysmal tachycardia	I47	1
	Atrial fibrillation and flutter	I48	
	Other cardiac arrhythmias	I49 (excluded I49.9)	
Celiac disease		K90.0	
Chronic interstitial pulmonary disease	Interstitial pulmonary fibrosis		1
	Long interstitial pulmonary diseases	J84	
Chronic kidney disease		N18	1
Congestive heart failure	Heart failure	I50	1
	Pulmonary edema	J81	
	Cardiomyopathy	I42 (excluded I42.6)	
Depression		F32	1
Diabetes Mellitus	Diabetes type1	E10	1
	Diabetes type2	E11	
Diseases of the nervous system	Trigeminal neuralgia	G50.0	1
	Neuropathies	G50, G51, G52, G60.9	
	Myoneural junction or muscle disease	G70, G73	
	Nerve root and plexus disorders	G54.9	
	Neuropathies	G56, G57, G58, G60, G62.81, G64	
Epilepsy		G40	1
Fatty Liver		K73, K75, K76	1
Hypertension		I10	1
Ischemic heart disease	Acute myocardial infarction	I21, I22, I24	1
	Angina pectoris	I20	
	Chronic ischemic heart disease	I25	
Parkinsonism		G20	1
Psychosis		F20, F22, F23, F24, F25, F28, F30	1
Pulmonary disease	Emphysema	J43	
	Chronic obstructive pulmonary disease	J44	
	Bronchiectasis	J47	

C. Acute diagnoses			
**Outcome**	**Subcategory**	**ICD-10**	**First occurrence**
Acute respiratory distress syndrome		J80	1
Appendicitis		K35	1
Cerebral hemorrhage		I60, I61, I62	1
Conjunctivitis		H10	
Diseases of the pleura	Pneumothorax	J93	1
	Other pleural condition	J94	
Encephalitis		G04, G05, A86, A85	
Guillain-Barré syndrome		G61	
Herpes simplex		B00	1
Hospitalization			
Hyperreactive airway disease	Bronchitis	J40, J41, J42	1
	Asthma	J45	1
Invasive pneumococcal		J13	1
Ischemic stroke and TIA		I63, G45	1
Kawasaki disease and<br>Pediatric inflammatory multisystem syndrome		M30.3	
Lung abscess		J85	1
Myocarditis		I40, I41	
Pericarditis	Acute pericarditis	I30	1
	Other diseases of pericardium	I31	
Pulmonary eosinophilia		J82	1
Respiratory failure		J96	1
Venous thromboembolism		I26, I80, I82	

D. Infectious diseases			
**Outcome**	**Subcategory**	**ICD-10**	**First occurrence**
EBV infection		*Labs - serology*	
Herpes Zoster		B02	
Streptococcal tonsillitis		*Labs - culture*	

Supplementary Table S3 (A-D). List of outcomes in the categories "Delayed acute complications" (A), "Chronic diseases" (B), "Persisting symptoms (hospital diagnoses)" (C), "Prescription drug use" (D) investigated by Lund et al. (6). Conditions that were not considered in comparative analyses between COVID-positive and COVID-negative subjects due to insufficient numbers of events are highlighted in gray.

**A. Delayed acute complications**		
**Outcome**	**Components**	**ICD-10**
Myocarditis	-	I40, I41, I54.1
Venous thromboembolism	Pulmonary embolism, deep vein thrombosis	I26, I80.1, I80.2, I80.3, I80.8, I80.9, I82.2, I82.3, I82.9
Peripheral vascular disease		
Arterial embolism and thrombosis	-	I73.9, I74, I77.9
Ischaemic stroke or TIA	-	I63, I64, G45
Cerebral haemorrhage	-	I60-I62
Guillian-Barré syndrome	-	G61.0
Encephalitis	-	G04, G05
Psychosis	-	F20-F29 (excluded F21)
Kawasaki disease	-	M30.3
PIMS-related diagnoses	-	M30.3, A48.3, I40, I41
Acute kidney injury	Defined using creatinine measurements according to KDIGO (Kidney Disease: Improving Global Outcomes) guidelines	

B. Chronic diseases		
**Outcome**	**Components**	**ICD-10**
Pulmonary disease	Asthma, chronic obstructive pulmonary disease, bronchiectasis, other chronic lower respiratory diseases, interstitial lung diseases, lung abscess, lung empyema, pneumothorax, pleural effusion	J40-J47, J80-J99
Interstitial pulmonary fibrosis	-	J84.1
Cardiovascular disease	Ischaemic heart disease, pulmonary embolism, pericarditis, myocarditis, supraventricular tachycardias, heart failure, ischaemic stroke	I20-I25, I26, I30-I32, I40-I43, I47-I50, I63, I64
Heart failure	-	I50
Diabetes mellitus	Type 1 diabetes, type 2 diabetes	E10-E14
Neurological disease	Encephalitis, myelitis, encephalomyelitis, epilepsy, migraine, other headache syndromes, neuropathies	G04, G05, G40, G43, G44, G45, G50-G59, G60-G64

C. Persisting symptoms (hospital diagnoses)		
**Outcome**	**Components**	**ICD-10**
Dyspnoea	-	R06.0
Cough	-	R05
Anosmia	-	R43.0
Headache	-	R51, G43, G44
Fatigue related disorders	-	R53, G93.3
Nonspecific pain	-	R52

D. Prescription drug use		
**Outcome**	**Components**	**Codes (ATC)**
Bronchodilating agents	Inhaled short- and long-acting beta-2 agonists, short- and long-acting muscarinic receptor antagonists	R03AC02-04, R03AC12-13, R03AC18-19, R03AK06-08, R03AK10-11, R03AL01-09, R03CC02, R03BB01, R03BB04-07
Short-acting beta-agonists (SABA)	-	R03AC02-04, R03AL01-02, R03CC02
Inhaled corticosteroids (ICS)	-	R03BA, R0AK, R03AL08, R03AL09
Cough preparations	Oral codeine and dextromethorphan	R05DA04, R05DA09
Paracetamol	-	N02BE01
NSAIDs	-	M01A (excluded M01AX)
Opiods and opiod-like drugs	-	N02A
Triptans	-	N02CC
Glucose lowering drugs	Oral antidiabetic drugs and insulin	A10
Antidepressants	-	N06A
Benzodiazapines and Z-drugs	Benzodiazepines and non-benzodiazepine benzodiazepine receptor agonists	N05BA, N05CF
Antipsychotics	First- and second-generation antipsychotics	N05A
Platelet inhibitors	Aspirin, clopidogrel, prasugrel, ticagrelor, dipyridamole	B01AC06, B01AC07, B01AC22, B01AC24, B01AC30, N02BA01, B01AC04
Anticoagulants	Direct-acting oral anticoagulants, vitamin-K antagonists	B01AA03, B01AA04, B01AF01-B01AF03, B01AE07

Supplementary Table S4. List of outcomes investigated by Lam et al. (7). Conditions that were not considered in comparative analyses between COVID-positive and COVID-negative subjects due to insufficient numbers of events are highlighted in gray.

**Disease**	**ICD-9-CM**
Myocardial infarction	410
Heart failure	428, 398.91, 402.01, 402.11, 402.91, 404.01, 404.03, 404.11, 404.13, 404.91, 404.93
Stroke	430-438
Atrial fibrillation	427.3
Coronary artery disease	410-414, 36.0, 36.1
Deep vein thrombosis	453
Interstitial lung disease	515, 516.3, 517.2, 517.8, 714.81, 135, 495
Acute respiratory distress syndrome	518.8x, 518.51-518.53
Chronic pulmonary disease transient	490-496
Chronic pulmonary disease	490-496
Seizure	333.2, 345, 649.4, 780.3, 779.0, 780.3
Bell's Palsy	351.0, 351.1, 351.8, 351.9
Encephalitis and Encephalopathy	323.0-323.9, 348.30, 348.31, 348.39
Anxiety	300.0
Post-traumatic stress disorder	308.x-309.x
Psychotic disorder	298.8, 298.9
Liver injury	570, 573.3
Pancreatitis	577.0, 577.1
Acute kidney injury	584.5-584.9
End stage renal disease	585.6, 586

Supplementary Table S5. List of outcomes investigated by Wan et al. (8).

Disease	ICD-10
Myocardial infarction	I21.x, I22.x, I23.x, I24.1, I25.2
Heart failure	I50.x
Pericarditis	I30, I31, I32, B33.23
Myocarditis	B33.20, B33.22, B33.24, I40.x, I51.4, I41.x
Stroke	I60.x, I61.x, I63.x, I64.x
TIA	G45.0, G45.1, G45.2, G45.8, G45.9
Atrial fibrillation	I48.x
Arrythmias, atrial flutter	I48.9, I48.3, I48.4
Coronary heart disease	I20.x - I25.x, I46.x
Acute coronary disease	I21.0- I21.3, I22.0, I22.1, I22.8, I21.4, I21.9, I22.9, I20.1, I20.8, I20.9, I20.0, I24.0, I24.8, I24.9
Deep vein thrombosis	I80-I81, I82.Ax, I82.Bx, I82.Cx
Superficial vein thrombosis	I82.8 I82.81x I82.89x

Supplementary Table S6. List of outcomes investigated by Horberg et al. (10), stratified by timing of onset.

	Time Period		
**CS PASC Related Conditions**	**Late**	**Acute and persistent**	**Pre-existing conditions**
Other lower respiratory disease	2.73%	4.50%	10.40%
Diabetes	2.25%	1.32%	9.85%
Gastrointestinal disease	4.19%	1.59%	2.89%
Conditions associated with dizziness or vertigo	4.06%	0.91%	3.06%
Abdominal pain	4.34%	0.71%	2.95%
Nonspecific chest pain	4.17%	1.42%	1.61%
Mental health	2.64%	0.61%	3.21%
Anxiety disorders	2.74%	0.86%	2.27%
Genitourinary symptoms and ill-defined conditions	3.45%	0.46%	1.78%
Malaise and fatigue	3.42%	1.02%	0.84%
Cardiac dysrhythmias	2.26%	1.22%	1.31%
Other nervous system disorders	1.75%	0.46%	1.04%
Respiratory failure, insufficiency, arrest	0.21%	2.69%	0.23%
Nausea and vomiting	1.66%	0.30%	0.54%
Fluid and electrolyte disorders	0.99%	0.85%	0.55%
Other nutritional, endocrine, and metabolic disorders	1.16%	0.17%	0.31%
Anosmia	0.73%	0.05%	0.01%

Supplementary Tables S7 (A-H). Comparison between outcomes and extraction criteria across studies. Conditions that were significantly different between the groups are in bold. Areas that did not reach a sufficient sample size for analysis are in gray. C = Chronic condition; A = Acute condition.

S7-A – CARDIOVASCULAR OUTCOMES

Mizrahi *et al.* - BMJ 2023				Lund *et al.* - The Lancet Infection 2021				Lam *et al.* - The Lancet 2023			Wan *et al.* - Cardiovascular Research 2023	
**Outcome**	**Subgroup**	**ICD-10**		**Outcome**	**Components**	**ICD-10**		**Outcome**	**ICD-9**	**ICD-10**	**Disease**	**ICD-10**
Cardiac arrhythmias	Paroxysmal tachycardia	I47	C					**Atrial fibrillation**	**427.3x**	**I48.x**	**Atrial fibrillation**	**I48.x**
	Atrial fibrillation and flutter	I48									**Arrythmias, atrial flutter**	**I48.9, I48.3, I48.4**
	Other cardiac arrhythmias	I49 (excluded I49.9)										
Congestive heart failure	heart failure	I50	C	Heart failure		I50	C	**Heart failure**	**428xx, 398.91, 402.01, 402.11, 402.91, 404.01, 404.03, 404.11, 404.13, 404.91, 404.93**	**I50.x**	**Heart failure**	**I50.x**
	Pulmonary edema	J81										
	Cardiomyopathy	I42 (excluded I42.6)										
Ischemic heart disease	Acute myocardial infarction	I21, I22, I24	C					**Coronary artery disease**	**410-414, 36.0, 36.1**	**I20.x - I25.x, I46.x**	**Coronary heart disease**	**I20.x - I25.x, I46.x**
	Angina pectoris	I20						Myocardial infarction	410	I21.x, I22.x, I23.x, I24.1, I25.2	**Myocardial infarction**	**I21.x, I22.x, I23.x, I24.1, I25.2**

Mizrahi *et al.* - BMJ 2023				Lund *et al.* - The Lancet Infection 2021				Lam *et al.* - The Lancet 2023			Wan *et al.* - Cardiovascular Research 2023	
**Outcome**	**Subgroup**	**ICD-10**		**Outcome**	**Components**	**ICD-10**		**Outcome**	**ICD-9**	**ICD-10**	**Disease**	**ICD-10**
	Chronic ischemic heart disease	I25									**Acute coronary disease**	**I21.0- I21.3, I22.0, I22.1, I22.8, I21.4, I21.9, I22.9, I20.1, I20.8, I20.9, I20.0, I24.0, I24.8, I24.9**
Cerebral hemorrhage		I60, I61, I62	A	Cerebral haemorrhage		I60-I62	A					
Ischemic stroke and TIA		I63, G45	A	Ischaemic stroke or TIA		I63, I64, G45	A	Stroke	430-438	I60.x, I61.x, I63.x, I64.x	**Stroke**	**I60.x, I61.x, I63.x, I64.x**
											TIA	G45.0, G45.1, G45.2, G45.8, G45.9
Myocarditis		I40, I41	A	Myocarditis		I40, I41, I541	A				Myocarditis	B33.20, B33.22, B33.24, I40.x, I51.4, I41.x
Pericarditis	Acute pericarditis	I30	A								Pericarditis	I30, I31, I32, B33.23
	Other diseases of pericardium	I31										
Venous thrombo-embolism		I26, I80, I82	A	**Venous thrombo-embolism**	**Pulmonary embolism, deep vein thrombosis**	**I26, I801, I802, I803, I808, I809, I822, I823, I829**	**A**	**Deep vein thrombosis**	**453**	**I80-I81, I82.Ax, I82.Bx, I82.Cx**	**Deep vein thrombosis**	**I80-I81, I82.Ax, I82.Bx, I82.Cx**
											Superficial vein thrombosis	I82.8 I82.81x I82.89x
**Hypertension**		**I10**										

Mizrahi *et al.* - BMJ 2023				Lund *et al.* - The Lancet Infection 2021				Lam *et al.* - The Lancet 2023			Wan *et al.* - Cardiovascular Research 2023	
**Outcome**	**Subgroup**	**ICD-10**		**Outcome**	**Components**	**ICD-10**		**Outcome**	**ICD-9**	**ICD-10**	**Disease**	**ICD-10**
				Cardiovascular disease	Ischaemic heart disease, pulmonary embolism, pericarditis, myocarditis, supra-ventricular tachycardias, heart failure, ischaemic stroke	I20-I25, I26, I30-I32, I40-I43, I47-I50, I63, I64	C					
				Peripheral vascular disease								
											Stable angina	
											**Unstable angina**	
											Ischemic cardiomyopaty	
											NIC	
											**Cardiac arrest**	
											**Cardiogenic shock**	

S7-B – RESPIRATORY OUTCOMES

Mizrahi *et al.* - BMJ 2023				Lund *et al.* - The Lancet Infection 2021				Lam *et al.* - The Lancet 2023		
**Outcome**	**Subgroup**	**ICD-10**		**Outcome**	**Components**	**ICD-10**		**Outcome**	**ICD-9**	**ICD-10**
Diseases of the pleura	Pneumothorax	J93	A							
	Other pleural condition	J94								
**Hyperreactive airway disease**	**Bronchitis**	**J40, J41, J42**	**A**							
	**Asthma**	**J45**								
Invasive pneumococcal		J13	A							
Respiratory failure		J96								
Pulmonary disease	Emphysema	J43	C							
	Chronic obstructive pulmonary disease	J44								
	Bronchiectasis	J47								
Chronic interstitial pulmonary disease	Interstitial pulmonary fibrosis		C	Interstitial pulmonary fibrosis		J84.1	C	**Interstitial lung disease**	**515, 516.3, 517.2, 517.8, 714.81, 135, 495**	**M05.1, J84, J81, J82**
	Long interstitial pulmonary diseases	J84								
Acute respiratory distress syndrome		J80	A					**Acute respiratory distress syndrome**	**518.8x, 518.51-518.53**	**J96.0, J96.10, J96.2, J80, R06.3, J98.4, J95.1, J95.2, J95.3, J95.821, J95.822**
Lung abscess		J85	A							
Pulmonary eosinophilia		J82	A							

Mizrahi *et al.* - BMJ 2023				Lund *et al.* - The Lancet Infection 2021				Lam *et al.* - The Lancet 2023		
**Outcome**	**Subgroup**	**ICD-10**		**Outcome**	**Components**	**ICD-10**		**Outcome**	**ICD-9**	**ICD-10**
				Pulmonary disease	Asthma, chronic obstructive pulmonary disease, bronchiectasis, other chronic lower respiratory diseases, interstitial lung diseases, lung abscess, lung empyema, pneumothorax, pleural effusion	J40-J47, J80-J99	C			
								Chronic pulmonary disease	490-496	J40.x-J47.x, J60.x-J67.x, J68.4, J70.1, J70.3

S7-C – NEUROLOGIC OUTCOMES

Mizrahi *et al.* - BMJ 2023				Lund *et al.* - The Lancet Infection 2021				Lam *et al.* - The Lancet 2023		
**Outcome**	**Subgroup**	**ICD-10**		**Outcome**	**Components**	**ICD-10**		**Outcome**	**ICD-9**	**ICD-10**
**Diseases of the nervous system**	**Trigeminal neuralgia**	**G50.0**	**C**							
	**Neuropathies**	**G50, G51, G52, G60.9**		Neuropathies		G50-59, G60-G64	C			
	**Myoneural junction or muscle disease**	**G70, G73**								
	**Nerve root and plexus disorders**	**G54.9**								
	**Neuropathies**	**G56, G57, G58, G60, G62.81, G64**								
Epilepsy		G40	C					**Seizure**	**333.2, 345, 649.4, 780.3, 779.0, 780.3**	**G40-G41**
Parkinsonism		G20	C							
Encephalitis		G04, G05, A86, A85	A	Encephalitis		G04, G05	A	Encephalitis and Encephalopathy	323.0-323.9, 348.30, 348.31, 348.39	G05.3, G04.9, G04.91, G93.41, I67.83
Guillain-Barré syndrome		G61		Guillian-Barré syndrome		G610	A			
				Neurological disease	Encephalitis, myelitis, encephalomyelitis, epilepsy, migraine, other headache syndromes, neuropathies	G04, G05, G40, G43, G44, G45, G50-G59, G60-G64	C			
								Bell's Palsy	351.0, 351.1, 351.8, 351.9	G51.1, G51.2, G51.4, G51.8, G51.9

S7-D – MENTAL HEALTH

Mizrahi *et al.* - BMJ 2023			Lund *et al.* - The Lancet Infection 2021				Lam *et al.* - The Lancet 2023		
**Outcome**	**ICD-10**		**Outcome**	**Components**	**ICD-10**		**Outcome**	**ICD-9**	**ICD-10**
**Anxiety**	**F41**	**C**	Anxiety disorders		F41 F43	C	**Anxiety**	**300.0**	**F40-F41**
Depression	F32	C	Depression		F32	C			
Psychosis	F20, F22, F23, F24, F25, F28, F30	C	Psychosis		F20-F29 (excluded F21)	A			
			**Psychiatric illness**	**Psychoses, affective disorders, neurotic- and stress disorders**	**F20-F29, F30-F39, F40-F48**	**C**			
							Psychotic disorder	298.8, 298.9	F23, F29
							**Post-traumatic stress disorder**	**308.x-309.x**	**F43.0, R45.7, F43.20, F43.21, F432.4, F43.25**

S7-E – METABOLIC

Mizrahi *et al.* - BMJ 2023				Lund *et al.* - The Lancet Infection 2021				Naveed *et al.* - AMA Network Open. 2023		
**Outcome**	**Subgroup**	**ICD-10**		**Outcome**	**Components**	**ICD-10**		**Outcome**	**ICD-9**	**ICD-10**
Diabetes Mellitus	Diabetes type1	E10	C	Diabetes mellitus	Type 1 diabetes, type 2 diabetes	E10-E14	C	**Diabetes mellitus**	**250.xx**	**E10-E14**
	Diabetes type2	E11								

S7-F – PEDIATRIC

Mizrahi *et al.* - BMJ 2023			Lund *et al.* - The Lancet Infection 2021		
**Outcome**	**ICD-10**	**Outcome group**	**Outcome**	**ICD-10**	
Kawasaki disease and Pediatric inflammatory multisystem syndrome	M30.3	A	Kawasaki disease	M30.3	A
			PIMS-related diagnoses	M30.3, A48.3, I40, I41	

S7-G – MISCELLANEOUS

Mizrahi *et al.* - BMJ 2023			Lund *et al.* - The Lancet Infection 2021			Lam *et al.* - The Lancet 2023		
**Outcome**	**ICD-10**		**Outcome**	**ICD-10**		**Outcome**	**ICD-9**	**ICD-10**
Celiac disease*	K90.0	C						
**Fatty Liver**	**K73, K75, K76**	**C**						
Appendicitis	K35	A						
**Conjunctivitis**	**H10**	**A**						
**Herpes simplex**	**B00**	**A**						
**Hospitalization**		**A**						
Chronic kidney disease	N18	C						
			Acute kidney injury	*Defined using creatinine measurements according to KDIGO guidelines*	A	**Acute kidney injury**	**584.5-584.9**	**N17, N19**
						**End stage renal disease**	**585.6, 586**	**I12.0. I13.x, N18.5, N18.6, Z99.2, N19.x, Z94.0, Z49.0**
						**Pancreatitis**	**577.0, 577.1**	**K85.0x, K85.8x, K85.9x**
						Liver injury	570, 573.3	K72.00, K76.2, K71.6, K75.9

S7-H – SYMPTOMS

Mizrahi *et al.* - BMJ 2023			Lund *et al.* - The Lancet Infection 2021	
**Outcome**	**Subgroup**	**ICD-10**	**Outcome**	**ICD-10**
Abdominal pain		R10.9		
**Anosmia and Dysgeusia**		**R43.0, R43.8**	Anosmia	R430
**Arthralgia**		**M25.5**		
Bloody stool		K92.1		
**Chest pain**		**R07.9, R07.1, R07.8, R07.2**		
Chills		R68.83		
**Concentration and memory impairment**	**Memory difficulties**	**R41.83, R41.2, R68.89**		
	**ADHD**	**F90.0 (314.00, Y23257)**		
	**Cognitive deficit**	**R41.83, R41.2**		
**Cough**		**R05**	**Cough**	**R05**
Convulsions		R56.9, R56.01, R56.00		
Diarrhea		R19.7		
**Dizziness**		**R42**		
**Dyspnea**		**R06**	**Dyspnoea**	**R060**
**Hair loss**		**L65.8, L65.0, L65.9**		
Headache		G44, G43	Headache	R51, G43, G44
Hyperhidrosis		R61		
Insomnia		F51		
Loss of appetite		R63.4, R63.6		
**Lymphadenopathy**		**R59.9**		
Menstruation abnormalities*		N91, N92		
**Muscle atrophy**		**M62.5**		
**Myalgia**		**M60.9**		
Nausea or vomiting		R11.0, R11.2, R11.10		

Mizrahi *et al.* - BMJ 2023			Lund *et al.* - The Lancet Infection 2021	
**Outcome**	**Subgroup**	**ICD-10**	**Outcome**	**ICD-10**
**Palpitations**		**R00.0, R00.2**		
Paresthesia		R20		
**Respiratory disorders**		**J98 (excluded J98.1, J98.5, J98.6, J98.8, J98.01)**		
Rhinorrhea		J34.8		
Skin rash		R21, R50.6, R53.9		
Sore throat		R07.0		
**Tinnitus**		**H93**		
Tremor		R25.9		
Visual disturbances		H53		
Voice disorder		R49.9		
**Weakness**		**R53**	Fatigue related disorders	R53, G93.3
Weight loss		R63.4		
			Nonspecific pain	R52

Funding

This research was funded by CARIPLO foundation as part of the “Networking, ricerca e formazione sulla sindrome post COVID” grant; project “The Post-Covid-19 Syndrome: network building and innovative management to address a new public health emergency”, ID. 2021-4388, PI Claudio Lucifora.

PASCNET (Post-Acute Sars-Cov-2 syndrome NETwork) study group composition

Claudio Lucifora (1), Daria Vigani (1,3), Federico Franzoni (1), Gabriele Letta (1), Laura Antolini (2), Giuseppe Lapadula (2), Elena Tassistro (2), Maria Grazia Valsecchi (2), Stefano Denicolai (3), Marica Grego (3), Diala Kabbara (3), Costanza Baldrighi (3), Antonio Giampiero Russo (4), Pietro Magnoni (4), Cristina Mazzali (4), Alberto Milanese (4), Rossella Murtas (4), Andrea Salvatori (4), Deborah Testa (4), Sara Tunesi (4), Adele Zanfino (4), Simona Dalle Carbonare (5), Federica Manzoni (5), Simona Migliazza (5), Pietro Giovanni Perotti (5), Linda Guarda (6), Marco Villa (6), Silvia Tillati (7), Giacomo Crotti (7), Giuseppe Sampietro (7), Alberto Zucchi (7), Anita Andreano (8), Luca Cavalieri d’Oro (8), Elisabetta Merlo (8), Magda Rognoni (8), Piersimone Fontana (9), Giovanni Maifredi (9), Ivan Cometti (10), Anna Clara Fanetti (10), Maria Letizia Gambino (11), Monica Lanzoni (11), Giuseppe Emanuele La Piana (12), Anna Bussi (13), Vincenzo Belcastro (14), Stefano Rusconi (15), Luigi Magnani (16), Maurizio Morlotti (17), Andrea Patroni (17), Raffaele Bruno (18), Elisabetta Pagani (18), Paolo Sacchi (18), Valentina Zuccaro (18).

(1) Catholic University of the Sacred Heart of Milan, Milan, Italy;
(2) University of Milano-Bicocca, School of Medicine and Surgery and Bicocca Bioinformatics Biostatistics and Bioimaging Centre (B4), Milan, Italy;
(3) Department of Economics and Management, University of Pavia, Pavia, Italy;
(4) Epidemiology Unit, Agency for Health Protection Milan, Milan, Italy;
(5) Epidemiology Unit, Agency for Health Protection Pavia, Pavia, Italy;
(6) Epidemiology Unit, Agency for Health Protection Val Padana, Cremona, Italy;
(7) Epidemiology Unit, Agency for Health Protection Bergamo, Bergamo, Italy;
(8) Epidemiology Unit, Agency for Health Protection Brianza, Monza, Italy;
(9) Epidemiology Unit, Agency for Health Protection Brescia, Brescia, Italy;
(10) Epidemiology Unit, Agency for Health Protection Montagna, Sondrio, Italy;
(11) Epidemiology Unit, Agency for Health Protection Insubria, Varese, Italy;
(12) Respiratory Rehabilitation Unit, ASST Crema – Ospedale Santa Marta Rivolta D’Adda, Crema, Italy;
(13) General Medicine Unit, ASST del Garda – Presidio Ospedaliero di Manerbio/Leno;
(14) Neurology Unit, ASST Lodi – Ospedale Maggiore di Lodi, Lodi, Italy;
(15) Infectious Diseases Unit, ASST Ovest Milanese – Ospedale di Legnano, Legnano, Italy;
(16) Internal Medicine Unit, ASST Pavia – Ospedale Civile di Voghera, Voghera, Italy;
(17) ASST Valcamonica, Brescia, Italy;
(18) Fondazione IRCCS Policlinico San Matteo, Pavia, Italy.
